# Selective laser trabeculoplasty versus 0·5% timolol eye drops for the treatment of glaucoma in Tanzania: a randomised controlled trial

**DOI:** 10.1016/S2214-109X(21)00348-X

**Published:** 2021-10-13

**Authors:** Heiko Philippin, Einoti Matayan, Karin M Knoll, Edith Macha, Sia Mbishi, Andrew Makupa, Cristóvão Matsinhe, Vasco da Gama, Mario Monjane, Awum Joyce Ncheda, Francisco Alcides Mulobuana, Elisante Muna, Nelly Fopoussi, Gus Gazzard, Ana Patricia Marques, Peter Shah, David Macleod, William U Makupa, Matthew J Burton

**Affiliations:** a International Centre for Eye Health, Faculty of Infectious and Tropical Diseases, London School of Hygiene & Tropical Medicine, London, UK; b MRC International Statistics and Epidemiology Group, London School of Hygiene & Tropical Medicine, London, UK; c Eye Department, Kilimanjaro Christian Medical Centre, Moshi, Tanzania; d Kilimanjaro Christian Medical University College, Moshi, Tanzania; e Eye Centre, Medical Centre-University of Freiburg, Faculty of Medicine, University of Freiburg, Freiburg im Breisgau, Germany; f University Hospitals Birmingham NHS Foundation Trust, Birmingham, UK; g Provincial Hospital of Pemba, Pemba, Mozambique; h Hospital Central de Quelimane, Quelimane, Mozambique; i Presbyterian Eye Hospital, Bafoussam, Cameroon; j Cameroon Baptist Convention Health Services, Douala, Cameroon; k NIHR Biomedical Research Centre for Ophthalmology, Moorfields Eye Hospital NHS Foundation Trust–University College London Institute of Ophthalmology, London, UK; l University College London Institute of Ophthalmology, London, UK; m Birmingham Institute for Glaucoma Research, Institute of Translational Medicine, University Hospitals Birmingham, Birmingham, UK; n Centre for Health and Social Care Improvement, University of Wolverhampton, Wolverhampton, UK

## Abstract

**Background:**

Glaucoma is a major cause of sight loss worldwide, with the highest regional prevalence and incidence reported in Africa. The most common low-cost treatment used to control glaucoma is long-term timolol eye drops. However, low adherence is a major challenge. We aimed to investigate whether selective laser trabeculoplasty (SLT) was superior to timolol eye drops for controlling intraocular pressure (IOP) in patients with open-angle glaucoma.

**Methods:**

We did a two-arm, parallel-group, single-masked randomised controlled trial at the Eye Department of Kilimanjaro Christian Medical Centre, Moshi, Tanzania. Eligible participants (aged ≥18 years) had open-angle glaucoma and an IOP above 21 mm Hg, and did not have asthma or a history of glaucoma surgery or laser. Participants were randomly assigned (1:1) to receive 0·5% timolol eye drops to administer twice daily or to receive SLT. The primary outcome was the proportion of eyes from both groups with treatment success, defined as an IOP below or equal to target pressure according to glaucoma severity, at 12 months following randomisation. Re-explanation of eye drop application or a repeat SLT was permitted once. The primary analysis was by modified intention-to-treat, excluding participants lost to follow-up, using logistic regression; generalised estimating equations were used to adjust for the correlation between eyes. This trial was registered with the Pan African Clinical Trials Registry, number PACTR201508001235339.

**Findings:**

840 patients were screened for eligibility, of whom 201 (24%) participants (382 eligible eyes) were enrolled between Aug 31, 2015, and May 12, 2017. 100 (50%) participants (191 eyes) were randomly assigned to the timolol group and 101 (50%; 191 eyes) to the SLT group. After 1 year, 339 (89%) of 382 eyes were analysed. Treatment was successful in 55 (31%) of 176 eyes in the timolol group (16 [29%] of 55 eyes required repeat administration counselling) and in 99 (61%) of 163 eyes in the SLT group (33 [33%] of 99 eyes required repeat SLT; odds ratio 3·37 [95% CI 1·96–5·80]; p<0·0001). Adverse events (mostly unrelated to ocular events) occurred in ten (10%) participants in the timolol group and in eight (8%) participants in the SLT group (p=0·61).

**Interpretation:**

SLT was superior to timolol eye drops for managing patients with open-angle high-pressure glaucoma for 1 year in Tanzania. SLT has the potential to transform the management of glaucoma in sub-Saharan Africa, even where the prevalence of advanced glaucoma is high.

**Funding:**

Christian Blind Mission, Seeing is Believing Innovation Fund, and the Wellcome Trust.

**Translations:**

For the Kiswahili, French and Portuguese translations of the abstract see Supplementary Materials section.

## Introduction

Glaucoma is a group of diseases that affect the optic nerve and lead to a progressive and irreversible loss of vision. Early stages of glaucoma can be asymptomatic, or individuals might notice missing or blurred areas in their field of vision.^1^ Late stages of the condition can lead to irreversible absolute blindness, particularly if left untreated. The main modifiable risk factor is elevated intraocular pressure (IOP); lifelong IOP control can halt disease progression.^2^, ^3^

Globally, glaucomas are the most frequent cause of irreversible blindness.^4^ Africa has the highest prevalence of glaucoma of all world regions, which is estimated to be 4·8%, as well as the highest incidence, with an expected increase from 10·31 million new cases in 2020 to 19·14 million in 2040 due to increasing life expectancy and population growth.^5^ The prevalence of blindness due to glaucoma is higher in sub-Saharan Africa than in any other world region.^4^ This situation is met by limited resources in many regions of sub-Saharan Africa; the mean number of ophthalmologists is 3·7 per million people in low-income countries versus 76·2 per million people in high-income countries.^6^

Reducing IOP by medical therapy with eye drops, surgery, or laser treatment is currently the only available treatment approach for delaying glaucoma progression. In sub-Saharan Africa, most people are either treated with the low-cost eye drops, timolol, or with surgery.^7^, ^8^ However, regular application of drops is often hampered by non-adherence, scarce availability, long-term costs, and side-effects. Trabeculectomy, the main surgical procedure for treating glaucoma, can effectively reduce IOP; however, the operation has a long learning curve, is offered in relatively few eye units across sub-Saharan Africa, can have clinically significant complications, and has a low uptake in some populations.^8^, ^9^, ^10^, ^11^, ^12^ Selective laser trabeculoplasty (SLT) is a rapid outpatient procedure used to reduce IOP. SLT increases aqueous fluid outflow from the eye, which drains through the trabecular meshwork. There is increasing evidence supporting its use as a primary intervention.^13^, ^14^ Lasers, especially SLT, could be part of future treatment for glaucoma in sub-Saharan Africa.^15^ However, to date, there have been no published trials of SLT in sub-Saharan Africa, and no trials worldwide have compared SLT with timolol as the standard treatment option.

**Research in context**

**Evidence before this study**

Preventing irreversible blindness from glaucoma can be achieved by reducing intraocular pressure (IOP) with daily eye drops, eye surgery, and laser treatment. African populations have the highest prevalence and incidence of open-angle glaucoma and the highest prevalence of blindness due to glaucoma worldwide. The *Lancet Global Health* Commission on global eye health called for research into cost-effective glaucoma interventions, especially those that are applicable in low-income and middle-income countries. Timolol eye drops are the most affordable and most commonly available treatment among drugs to reduce IOP. However, erratic application, systemic and local side-effects, and high long-term costs led to a search for alternatives methods to reduce IOP, such as selective laser trabeculoplasty (SLT). We did several literature searches using MEDLINE (via PubMed), ISRCTN, and PACTR trial registries, without any language or date restrictions, between Oct 3, 2012, and March 16, 2015, for published or ongoing trials of SLT as an alternative to timolol. Applying the terms “selective laser trabeculoplasty” (or “SLT”) and “timolol” showed no results. A wider search using “selective laser trabeculoplasty” (or “SLT”) without “timolol” found five randomised controlled trials that compared SLT with more expensive eye drops in different settings and reported SLT to be a feasible and safe alternative to medical ocular treatment.

**Added value of this study**

This trial was done in Tanzania and enrolled 201 patients with predominantly advanced glaucoma, reflecting the typical spectrum and associated challenges of glaucoma care in this region. By contrast, most patients in the previous trials involving SLT and more expensive eye drops had early or moderate glaucoma. Both eyes were enrolled if eligible and analysed using statistical methods that considered the correlation between the two eyes of a participant. This methodology efficiently used all available data, saving time and resources, and making optimal use of participants' engagement. 12 months after randomisation, the estimated odds for success using SLT were 3·37 times higher than those for success using 0·5% timolol eye drops. The odds ratio was not modified by other factors. Mean IOP reduction was 1·5 mm Hg (SD 7·5) in the timolol group and 6·3 mm Hg (6·4) in the SLT group between the baseline visit and visits at failure, success at 1 year, or before loss to follow-up. Safety, acceptance, vision-related quality of life, and preservation of visual acuity were similar in both groups after 1 year. Eye care units in the region would need to treat around 500 eyes per year with SLT to cover the cost of the procedure, which would cost an amount similar to a 1-year supply of timolol eye drops.

**Implications of all the available evidence**

This trial adds to the existing evidence that SLT is an important addition to the treatment options for glaucoma, and extends this evidence to regions where advanced glaucoma is more common and treatment resources and options are limited. The prevalence of glaucoma is expected to increase in the coming decades due to increasing life expectancy and population growth, especially in low-income and middle-income regions. Therefore, SLT could help to prevent vision loss and blindness from glaucoma in regions where its prevalence is highest.

We aimed to investigate whether SLT was superior to timolol eye drops for controlling IOP in patients with open-angle glaucoma in a Tanzanian setting.

## Methods

### Study design

We did a two-arm, parallel-group, single-masked randomised controlled trial at the Eye Department of Kilimanjaro Christian Medical Centre (KCMC), Moshi, northern Tanzania.

The trial was approved by the research ethics review committees of the National Institute for Medical Research in Dar es Salaam, Tanzania (NIMR/HQ/R.8a/Vol IX/1929), the Kilimanjaro Christian Medical University College in Moshi, Tanzania (number 800), and the London School of Hygiene & Tropical Medicine in London, UK (LSHTM Ethics Ref 7166). The trial was done in compliance with the Declaration of Helsinki and the International Conference on Harmonisation–Good Clinical Practice. An independent data and safety monitoring board was appointed by the trial steering committee. A patient steering group provided input on different aspects of the trial such as study design and questionnaires.

### Participants

Patients who attended the ophthalmology clinic at KCMC were screened for eligibility. The main inclusion criterion was diagnosis of chronic high-pressure open-angle glaucoma, defined as an IOP of more than 21 mm Hg and a combination of structural and functional changes (category 1 of the International Society of Geographic and Epidemiologic Ophthalmology).^16^ Structural changes were specified as thinning of the optic nerve head rim (stage 5 or above on the Disc Damage Likelihood Scale, a cup-to-disc ratio of ≥0·7, or a cup-to-disc ratio asymmetry between two eyes of ≥0·2).^17^ Functional changes included a glaucomatous visual field defect or relative afferent pupil defect. Inclusion criteria also permitted high-risk glaucoma suspect (IOP >25 mm Hg, structural changes as above, no visual field defect) or high-risk ocular hypertension (IOP >32 mm Hg, no structural or functional defect), and International Society of Geographic and Epidemiologic Ophthalmology category 2 (cup-to-disc ratio of ≥0·8 or cup-to-disc ratio asymmetry of ≥0·3 if a visual field could not be satisfactorily completed).^16^ Exclusion criteria included being aged younger than 18 years or having an opaque cornea, narrow angle (<2 on the Shaffer scale in two quadrants), absolute blindness (no perception of light), history of previous uveitis, any previous glaucoma surgery or laser treatment, neovascular or traumatic glaucoma, and history of asthma or bradycardia, which can be exacerbated by timolol eye drops. The full exclusion criteria are listed in appendix 4 (p 2). Patients who reported using eye drops before the trial had a 4-week washout period. Eligible patients were informed about the study in detail in Kiswahili and, if interested, invited to return on a different day for the baseline examination. During this assessment, written informed consent was obtained in Kiswahili before participants were enrolled.

### Randomisation and masking

The randomisation sequence was generated by an independent statistician with a variable block size between 4 and 8. Sequentially numbered and sealed opaque envelopes contained the allocation of participants to either the SLT or the timolol group (1:1). One or both eyes were enrolled, depending on eligibility, and were treated identically. Participants were enrolled and assigned to an intervention arm together by at least two of the following individuals: HP, EdM, SM, KMK, and EiM. Due to the nature of the interventions, participants, principal investigators, and health-care staff administering treatments could not be masked to treatment allocation; however, the clinicians who examined IOP were masked to the trial arm, the individual IOP threshold, and previous IOP measurements of the participant, and were not involved in any other aspect of the trial.

### Procedures

During the baseline assessment, a detailed clinical history was taken. We assessed vision-related quality of life using the 20-item cross-cultural WHO visual functioning questionnaire (WHO/PBD-VF20).^18^ Additional questionnaires included the Patient Outcome and Experience Measure and the Glaucoma Symptom Scale.^19^, ^20^

Visual acuity was measured at 2 m in a dimmed room (Peek Acuity app [version 3.5.0]). Static visual field perimetry was done with the Swedish interactive threshold algorithm standard 24-2 or 10-2 programme (II-i series system software version 4.2) of the Humphrey HFA II 740i Visual Field Analyzer (Carl Zeiss Meditec AG, Jena, Germany).^21^ The Disc Damage Likelihood Scale was used to stratify glaucoma severity into moderate (stage 5–7) and advanced (stage 8–10). Glaucoma-related structural features were assessed by slit-lamp examination of the anterior eye segment, pachymetry (central corneal thickness), gonioscopy, fundus imaging, and indirect fundoscopy of the optic nerve head.

Standardised examiners measured baseline IOP before treatment allocation, following a standard operating procedure. This procedure included measuring IOP with a calibrated Goldmann Applanation Tonometer (Haag Streit, Koeniz, Switzerland) twice within 5 mins. If the difference between the first two measurements was up to 2 mm Hg, the mean IOP was noted. Otherwise, a third measurement was obtained and the median was recorded.^22^ The repeatability coefficient of Goldmann tonometry is around 2·5 mm Hg.^23^

Several focus group discussions involving patients, relatives, and eye care specialists were held on the two treatment options and other contextual factors for glaucoma during the trial. The results from the questionnaires, focus group discussions, and other glaucoma-related functional or structural changes will be reported elsewhere.

Following the baseline assessment and enrolment, patients randomly assigned to the SLT laser intervention (SLT group) received amethocaine (topical anaesthesia), 0·2% topical brimonidine (IOP spike prevention), and 1·0% topical prednisolone (inflammatory response control) 15 mins before the procedure. The chamber angle was then visualised with the Latina goniolens supplied with the SLT laser (Lumenis Selecta II Lumenis, Yokneam, Israel). Approximately 100 laser spots were applied to cover 360° of the trabecular meshwork. Starting energy level was 0·6 mJ, which was continuously titrated in steps of 0·1 mJ until cavitation bubbles appeared in around a third of laser spot applications. The eye, including IOP, was examined about 1 h after SLT. All SLT procedures were done on the day of treatment allocation by one ophthalmologist (HP), who was trained in the procedure at University Hospitals Birmingham (Birmingham, UK) by PS and had completed around 100 SLT procedures before the trial.

Participants randomly assigned to the standard treatment arm (timolol group) received 0·5% timolol eye drops to administer twice daily. The importance, side-effects, and application of eye drops were explained by a study assistant to participants and accompanying helpers in Kiswahili using a standard protocol (appendix 4 pp 12–13). Adherence was estimated by asking participants at each follow-up visit how frequently they had missed their eye drops. Both treatment options were provided free of charge to the patient, and transport cost was subsidised to further increase adherence.^8^, ^24^

Follow-up assessments were scheduled at 2, 6, 9, and 12 months. Masked examiners measured IOP on each follow-up visit following the same procedure as was used during baseline assessment. Additional safety visits were arranged if the supervising clinician considered this to be necessary. One IOP measurement of up to 2 mm Hg above target IOP was allowed on one of the follow-up visits without triggering a repeat intervention or being considered a treatment failure. If the IOP was more than 2 mm Hg above target or up to 2 mm Hg above target for the second time, repeat SLT or counselling was provided. If the IOP exceeded the target on any subsequent occasion again, the eye was considered to have a treatment failure and exited from the trial, and the patient received additional treatment (including the intervention from the other intervention arm, additional eye drops, or trabeculectomy; appendix 4 p 4). Furthermore, if the IOP was more than 40 mm Hg at any visit, the eye was considered to have a treatment failure and was exited from the trial immediately; additional treatment was provided to the participant's eye.

To estimate the cost of an SLT laser procedure, we followed a bottom-up micro-costing approach assuming that the equipment had a lifetime of 10 years and that the SLT treatment was done on demand during a glaucoma clinic by an ophthalmologist earning a standard salary.^13^, ^25^ Variable and fixed costs were calculated and a threshold analysis was done estimating total costs for eight production scenarios, depending on the annual number of treatments (appendix 4 pp 10–12). The annual cost of timolol eye drops was identified using the median of three prices at pharmacies across Tanzania and was used as a reference to determine the number of SLT procedures that would result in comparable cost. Both annual treatment costs were then compared with an affordability threshold of 2·5% of Tanzania's gross domestic product per capita, as a proxy of income.^26^

### Outcomes

The primary outcome was the proportion of eyes from both intervention groups with treatment success at 12 months following randomisation. For patients with advanced glaucoma (stage 8–10 according to the Disc Damage Likelihood Scale), this target IOP was 18 mm Hg or below and for those with moderate glaucoma (stage 5–7), this target was 21 mm Hg or below. Secondary outcomes were safety, acceptance, vision-related quality of life, adherence, preservation of visual acuity and visual fields, other glaucoma-related functional or structural changes, other IOP-related outcomes, analyses of focus group discussions, cost, and treatment affordability.

### Statistical analysis

The trial was powered to test the hypothesis that SLT is superior to timolol eye drops. From the literature and retrospective data from the Eye Department at KCMC, we anticipated that the proportions of success after 12 months would be 60% for timolol and 75% for SLT.^27^ Allowing for a loss to follow-up of 20%, a sample size of 360 eyes was estimated to provide 80% power with 95% confidence to detect such a difference.

The primary outcome was a binary variable defined as treatment success at 12 months, compared between the two treatment arms. Analysis of the primary outcome was by modified intention-to-treat using a logistic regression model, in which participants lost to follow-up were excluded, with generalised estimating equations (GEE) to account for the absence of independence between eyes, if both eyes were included. The primary analysis was unadjusted, although baseline characteristics were examined for balance between arms.

Secondary outcomes were described and compared between the two treatment arms. A change in visual acuity of two or more lines on the logarithm of the minimum angle of resolution (logMAR) chart (equals ≥0·2 logMAR between baseline and the last visit, either in the event of a failure, before loss to follow-up, or success at 12 months) was defined as a loss of central vision and compared using logistic regression with GEE by arm.^28^ Acceptance was described as the number of times a participant refused an intervention at any of the follow-up visits after being randomly assigned. WHO/PBD-VF20 items were divided into the general functioning, visual symptoms, and psychosocial subscales, and summary scores were transformed to a scale (0–100), with 100 as the highest possible vision-related quality of life score.^18^ Affordability was described as whether a person had sufficient income to pay for health-care services, treatment, or costs (appendix 4 p 12).^29^

We tested for evidence of effect modification by the stage of glaucoma and baseline IOP. A sensitivity analysis was done to provide the most conservative estimate, considering all participants lost to follow-up as failure in the more successful arm and as success in the less successful arm. Patients lost to follow-up were compared with those who completed the trial with respect to age, sex, stage of glaucoma, intervention, visual field defect, visual acuity, and travel details using logistic regression with GEE. Differences between arms in time to an event were assessed by plotting survival curves and a Cox regression analysis, by use of a shared frailty model to account for dependency between the two eyes. Other potential determinants of success were investigated using logistic regression with GEE. To prevent multicollinearity in a fully adjusted model, all potential determinants were first screened for inclusion using a univariable model and GEE. Any factor in which p<0·2 was included in the fully adjusted model. Backward stepwise selection was then employed to find the most parsimonious logistic regression model, with p<0·05 for all predictors.

Data were managed in a custom built database in Microsoft Access 2016. Stata (version 16.1) was used to compute the statistical analysis. A data safety monitoring board oversaw the study. This trial was registered with the Pan African Clinical Trials Registry, number PACTR201508001235339.

### Role of the funding source

The funders had no role in study design, data collection, data analysis, data interpretation, or writing of the report.

## Results

**Figure 1:**
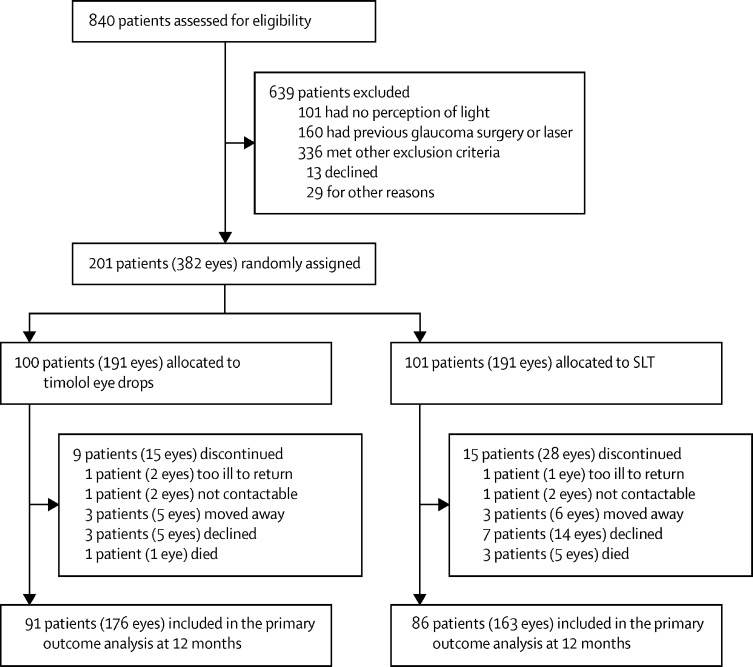
Trial profile SLT=selective laser trabeculoplasty. The full list of reasons for exclusion are provided in appendix 4 (p 3).

840 patients with glaucoma who attended the Eye Department at KCMC were screened for eligibility (figure 1; appendix 4 p 3). Of those screened, 201 (24%) eligible participants (382 eyes) were enrolled between Aug 31, 2015, and May 12, 2017, of whom 100 (50%) of participants (191 eyes) were randomly assigned to the timolol group and 101 (50%; 191 eyes) to the SLT group. All participants were members of one of the ethnic groups living in Tanzania (table 1). At 12 months, 177 (88%) patients (339 eyes) were included in the analysis; 24 (12%) patients (43 eyes) had been lost to follow-up. The mean age of 201 people enrolled in the trial was 66·3 years (SD 11·6) and 83 participants were female. The mean age of 639 patients not enrolled was 65·0 years (15·5) and 268 participants were female.

**Table 1:** Baseline participant and ocular characteristics

		**Timolol group**	**SLT group**
**Participant characteristics**			
Number of participants		100	101
Age, years		65·09 (10·79)	67·40 (12·33)
Sex			
	Female	46 (46%)	37 (37%)
	Male	54 (54%)	64 (63%)
Ethnic group			
	Chagga	54 (54%)	57 (56%)
	Pare	18 (18%)	23 (23%)
	Meru	4 (4%)	4 (4%)
	Maasai	4 (4%)	1 (1%)
	Sambaa	3 (3%)	2 (2%)
	Other	17 (17%)	14 (14%)
Education			
	Less than secondary level	70 (70%)	63 (62%)
	Secondary level or higher	30 (30%)	38 (38%)
Family history of glaucoma*			
	No	76 (76%)	77 (76%)
	Yes	24 (24%)	24 (24%)
Travel distance, km			
	<50	51 (51%)	54 (53%)
	≥50	49 (49%)	47 (47%)
**Ocular characteristics**			
Number of eyes		191	191
Visual acuity (logMAR)		0·48 (0·69)	0·49 (0·66)
Visual acuity (WHO categories)			
	Normal vision	147 (77%)	145 (76%)
	Low vision	20 (10%)	23 (12%)
	Blind	24 (13%)	23 (12%)
Exfoliation glaucoma			
	No	167 (87%)	166 (87%)
	Yes	24 (13%)	25 (13%)
Pseudophakia			
	No	185 (97%)	177 (93%)
	Yes	6 (3%)	14 (7%)
Vertical cup-to-disc-ratio		0·85 (0·15)	0·84 (0·16)
Intraocular pressure, mm Hg		26·96 (7·52)	26·38 (6·28)
Optic nerve head damage (DDLS)			
	5	34 (18%)	42 (22%)
	6	20 (10%)	22 (12%)
	7	25 (13%)	19 (10%)
	8	47 (25%)	40 (21%)
	9	33 (17%)	33 (17%)
	10	32 (17%)	35 (18%)
Stage of glaucoma (DDLS)			
	Moderate (stage 5–7)	79 (41%)	83 (43%)
	Advanced (stage 8–10)	112 (59%)	108 (57%)
Visual field (24-2), mean defect, dB†		−18·29 (11·09)	−16·02 (10·94)
Visual field (10-2), mean defect, dB†		−33·92 (0·58)	−30·71 (4·40)
Central corneal thickness, μm‡		522·89 (34·79)	519·16 (34·51)
Previous timolol eye drops§			
	No	83 (43%)	93 (49%)
	Yes	108 (57%)	98 (51%)

Data are mean (SD) or n (%), unless otherwise indicated. SLT=selective laser trabeculoplasty. logMAR=logarithm of the minimum angle of resolution. DDLS=Disc Damage Likelihood Scale.

* In a first-degree relative.

† Visual field examinations: 347 eyes completed 24-2 (175 in timolol group *vs* 172 in SLT group); eight eyes completed 10-2 only (four in timolol group *vs* four in SLT group); no visual field possible in 27 eyes (12 in timolol group *vs* 15 in SLT group) due to reduced central vision.

‡ Central corneal thickness measurements missing in 13 eyes (five in timolol group *vs* eight in SLT group) due to temporary failure of the pachymeter.

§ Based on patient history.

Loss to follow-up was not associated with age, sex, stage of glaucoma, intervention arm, or level of visual acuity. There was evidence that patients with advanced visual field defects were less likely (p=0·0018) and patients who needed a guide for their journey to the eye hospital were more likely (p=0·016) to be lost to follow-up. However, these inferences are based on few patients who were lost to follow-up (24 patients [12%]; figure 1).

**Figure 2:**
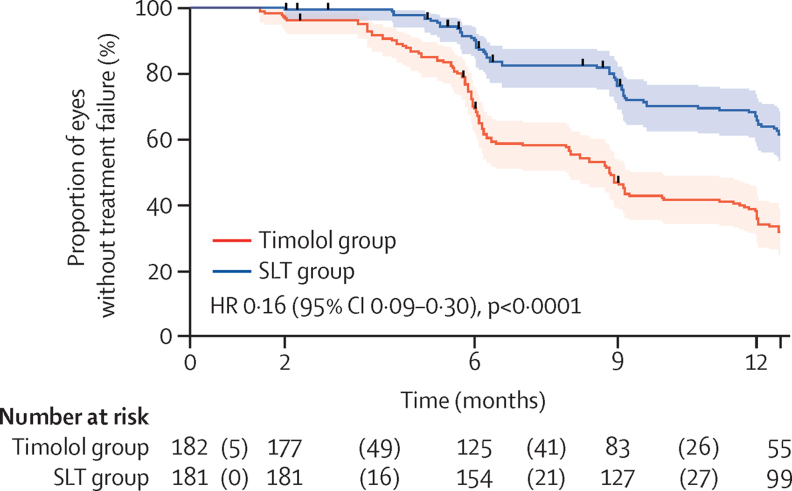
Kaplan-Meier curve of time to treatment failure Differences between the two intervention groups in time to an event was assessed with a Cox regression analysis using a shared frailty model to account for dependency between the two eyes. HR=hazard ratio. SLT=selective laser trabeculoplasty.

A successful IOP reduction 1 year after the start of treatment was reported in 55 (31%) of 176 eyes in the timolol group (16 [29%] of 55 eyes required repeat counselling) and in 99 (61%) of 163 eyes in the SLT group (33 [33%] of 99 eyes required a repeat SLT). The unadjusted logistic regression model (ie, GEE) for the relationship between intervention and success estimated an odds ratio (OR) of SLT over timolol eye drops of 3·37 (95% CI 1·96–5·80; p<0·0001; table 2). Cox regression analysis showed a hazard ratio of 0·16 (0·09–0·30; p<0·0001; figure 2). Detailed IOP results can be found in the appendix 4 (pp 5–6).

**Table 2:** Predicted ORs for success

	**Success**	**Univariable OR (95% CI)**	**p value**	**Multivariable OR (95% CI)**	**p value**
**Intervention**					
Timolol	55/176 (31%)	1 (ref)	..	..	..
SLT	99/163 (61%)	3·37 (1·96–5·80)	<0·0001	5·35 (2·77–10·31)	<0·0001
**Sex**					
Female	66/142 (46%)	1 (ref)	..	..	..
Male	88/197 (45%)	0·92 (0·54–1·57)	0·77	..	..
**Age groups, years**					
<70	101/211 (48%)	1 (ref)	..	..	..
≥70	53/128 (41%)	0·74 (0·43–1·28)	0·28	..	..
**Education**					
Less than secondary level	92/225 (41%)	1 (ref)	..	..	..
Secondary level or above	62/114 (54%)	1·68 (0·97–2·93)	0·066	..	..
**Travel distance to KCMC, km**					
<50	87/185 (47%)	1 (ref)	..	..	..
≥50	67/154 (44%)	0·86 (0·51–1·46)	0·58	..	..
**History of timolol eye drops**					
No	75/151 (50%)	1 (ref)	..	..	..
Yes	79/188 (42%)	0·73 (0·44–1·22)	0·24	..	..
**Pseudophakia**					
Phakic	145/324 (45%)	1 (ref)	..	..	..
Pseudophakic	9/15 (60%)	1·16 (0·41–3·29)	0·78	..	..
**Exfoliation glaucoma**					
No	147/297 (49%)	1 (ref)	..	..	..
Yes	7/42 (17%)	0·16 (0·06–0·44)	0·0004	0·16 (0·05–0·46)	0·0009
Central corneal thickness, μm*					
<520	67/164 (41%)	1 (ref)	..	..	..
≥520	85/172 (49%)	1·43 (0·88–2·33)	0·15	..	..
**Angle pigmentation**					
Light pigmentation	132/289 (46%)	1 (ref)	..	..	..
Strong pigmentation	22/50 (44%)	1·06 (0·53–2·14)	0·87	..	..
**Stage of glaucoma (DDLS)**					
Moderate (stage 5–7)	108/145 (74%)	1 (ref)	..	..	
Advanced (stage 8–10)	46/194 (24%)	0·14 (0·09–0·23)	<0·0001	0·11 (0·06–0·20)	<0·0001
**Intraocular pressure, mm Hg**					
<25	100/153 (65%)	1 (ref)	..	..	
≥25	54/186 (29%)	0·27 (0·17–0·44)	<0·0001	0·33 (0·19–0·60)	0·0003
**Visual acuity (WHO categories)**					
Normal vision	135/263 (51%)	1 (ref)	..	..	..
Low vision	12/33 (36%)	0·64 (0·33–1·25)	..	..	..
Blind	7/43 (16%)	0·38 (0·21–0·71)	0·0060†	..	..
**Glaucoma categories**					
Early	63/81 (78%)	1 (ref)	..	..	..
Moderate	31/46 (67%)	0·46 (0·23–0·94)	..	..	..
Advanced	15/35 (43%)	0·33 (0·15–0·73)	..	..	..
Severe	40/150 (27%)	0·13 (0·07–0·23)	..	..	..
End stage	5/27 (19%)	0·10 (0·04–0·27)	<0·0001†	..	..

Data are n/N (%). Results of 339 eyes analysed at 12 months using univariable and multivariable analyses of potential factors associated with success using logistic regression with general estimating equations. Parameters with p<0·2 in the log likelihood ratio test were included in the initial multivariable model. Backward stepwise selection was then employed to find the most parsimonious logistic regression model, in which all predictors had p<0·05. This final model included intervention, intraocular pressure at baseline, stage of glaucoma, and exfoliation glaucoma. OR=odds ratio. SLT=selective laser trabeculoplasty. KCMC=Kilimanjaro Christian Medical Centre. DDLS=Disc Damage Likelihood Scale.

* Central corneal thickness missing for three eyes.

† Wald test for trend.

A reduction of central vision occurred in 36 (19%) of 187 eyes in the timolol group and in 40 (21%) of 188 in the SLT group. There was no evidence of a difference between interventions (OR 1·16 [95% CI 0·66–2·06]; p=0·60). Vision-related quality of life measured with the WHO/PBD-VF20 showed no differences between the two groups (table 3).

**Table 3:** Vision-related quality of life

	**Timolol group (n=28)**	**SLT group (n=50)**	**Estimated group Δ (95% CI)**	**p value**
**General functioning**				
Baseline visit	79·5 (17·7)	72·5 (21·2)	1·91 (−6·17 to 10·00)	..
12-month visit/Δ (SD)	88·2 (15·6)/8·6 (19·7)	83·1 (15·8)/10·5 (15·6)	..	0·64
**Visual symptoms**				
Baseline visit	66·4 (17·3)	68·3 (21·9)	−2·67 (−11·89 to 6·55)	..
12-month visit/Δ (SD)	74·7 (16·9)/8·3 (23·8)	74·0 (16·9)/5·7 (16·9)	..	0·57
**Psychosocial**				
Baseline visit*	77·2 (17·3)	74·4 (21·1)	−5·29 (−15·02 to 4·44)	..
12-month visit/Δ (SD)	87·5 (17·3)/10·3 (15·2)	79·3 (24·2)/5·0 (23·1)	..	0·28

Data are mean (SD), unless otherwise indicated. Mean (SD) of total scores (0–100) of three subscales of WHO/PBD-VF20 questionnaires for patients with success at 12 months (if both eyes were enrolled, the status of the right eye was considered), so 28 patients in the timolol group and 50 patients in the SLT group. Higher scores represent a better vision-related quality of life. Estimated group difference (Δ), 95% CI, and p values from linear regression of differences between interventions. SLT=selective laser trabeculoplasty. Δ=delta or difference.

* Data of one patient missing in the SLT group.

Self-reported adherence to eye drop use in the timolol group was high (table 4). 56–75% of patients reported daily application of eye drops during the 2 weeks before the follow-up visit, 15–24% of patients reported missing eye drops for 1–2 days, and only 4–20% of patients reported missing eye drops for more than 2 days. No participant refused either timolol eye drops or SLT within the first year, including repeat interventions.

**Table 4:** Self-reported adherence to eye drops for participants in the timolol group

	**2-month visit (n=95)**	6-month visit (n=90)*	**9-month visit (n=51)**†	**12-month visit (n=36)**
Adherence every day	53 (56%)	62 (69%)	35 (69%)	27 (75%)
Non-adherence for 1–2 days	23 (24%)	13 (14%)	10 (20%)	7 (19%)
Non-adherence for >2 days	19 (20%)	14 (16%)	2 (4%)	2 (6%)

Adherence to treatment was assessed at each follow-up by asking participants how frequently they took their eye drops during the previous 2 weeks. Assessment continued for participants until the respective study exit (eg, failure, loss to follow-up, or success at 12 months).

* One reply missing.

† Three replies missing.

From an eye care provider's perspective, the variable cost per SLT treatment was estimated to be US$2·57. Annual fixed costs were $4960, including the depreciation of the initial purchase over the 10 years, the annual inspection, and an assumption of two repairs.^13^ Travel expenses of technicians were added, which might be substantial where services are not available in a country (appendix 4 pp 10–12). The SLT laser has been in operation at KCMC since 2015 without needing repair. With a scenario of 500 eyes treated per year, the total costs for one procedure are approximately $12·49. Since both eyes are often treated, this figure corresponds to 250–400 patients treated per year to cover the costs and offer the laser treatment at a price of $12·49 per treatment using a not-for-profit eye care service model (appendix 4 p 11). To achieve successful treatment with SLT in this study, 33 eyes required two procedures and 66 eyes were treated after one treatment. Thus, from the patient's perspective, an average of 1·33 treatments would be required, increasing the average cost to approximately $16·61 per eye for a successful outcome, excluding travelling expenses. Annual therapy with timolol eye drops cost around $16·32 per eye in Tanzania. Therefore, with around 500 treatments per year, the SLT treatment can be offered, covering costs, at a similar price as timolol eye drops. The annual GDP per capita in Tanzania in 2019 was reported to be $1122·12, so any annual treatment cost below $28·05 can be considered affordable. Thus, the annual treatment cost of timolol and SLT for one eye are below this threshold (assuming 500 procedures per year in an eye health unit). For SLT, the treatment costs for two eyes can also be considered affordable for most patients as 66 (67%) of 99 eyes only required one treatment for a successful outcome (annual treatment cost for two eyes of $24·98).

We used a sensitivity analysis to assess whether the primary outcome results were possibly influenced by loss to follow-up. The hypothetical scenario considered all patients who were lost to follow-up in the SLT group to be failures and those in the timolol group to be successes, assuming the worst possible scenario for the SLT group. The OR of success of SLT was 1·88 (95% CI 1·13–3·11; p=0·015).

There was no evidence of an effect modification in the OR of SLT over timolol by the stage of glaucoma (p=0·55) or by the baseline IOP (p=0·14; appendix 4 p 8).

Other potential determinants for success were evaluated (table 2). The most parsimonious multivariable model showed an association between success and SLT (*vs* timolol) as the randomisation arm (OR 5·35 [95% CI 2·77–10·31]; p<0·0001), high (*vs* low) IOP at baseline (0·33 [0·19–0·60]; p=0·0003), advanced (*vs* moderate) stage of glaucoma (0·11 [0·06–0·20]; p<0·0001), and the presence (*vs* absence) of exfoliation material (0·16 [0·05–0·46]; p=0·0009).

In total, there were ten (10%) ocular and systemic adverse events in the timolol group and eight (8%) in the SLT group (OR 0·77 [95% CI 0·29–2·05]; p=0·61; table 5). Four patients died during the 1-year follow-up period (one in the timolol group *vs* three in the SLT group) from known pre-existing general medical conditions. SLT was associated with several transient (<1 h) side-effects (appendix 4 p 9). The baseline SLT procedure caused no pain during 69 (36%) of 191 baseline laser procedures, mild pain during 103 (54%), moderate pain during 15 (8%), and severe pain during one (<1%). No baseline SLT procedure triggered an IOP spike of more than 5 mm Hg within the first hour, and two (2%) of 104 repeat SLT procedures were followed by reversible IOP spikes.

**Table 5:** Adverse events

		**Timolol group (n=100)**	**SLT group (n=101)**	**All (n=201)**
Total		10 (10%)	8 (8%)	18 (9%)
Ocular				
	Conjunctiva injected	2 (2%)	1 (1%)	3 (1%)
	Persistent cells in anterior chamber, hyphaemia	0	0	0
Systemic*				
	Cardiovascular event	1 (1%)	1 (1%)	2 (1%)
	Diabetes	3 (3%)	1 (1%)	4 (2%)
	Orthopaedic condition	2 (2%)	2 (2%)	4 (2%)
	Prostate surgery	1 (1%)	0	1 (<1%)
Death		1 (1%)	3 (3%)	4 (2%)

Data are n (%).

* Requiring hospital admission.

## Discussion

This randomised controlled trial compared timolol eye drops with SLT in patients with glaucoma in Tanzania. SLT was superior to timolol in controlling IOP, with an OR of 3·37 in favour of SLT (95% CI 1·96–5·80; p<0·0001). This difference between the two interventions was not significantly modified by the stage of glaucoma or baseline IOP.

A previous meta-analysis estimated the mean difference in IOP reduction between timolol and placebo at 3 months as 3·70 mm Hg (95% CI 3·16–4·24).^30^ We observed a comparable IOP reduction in the timolol group of 3·22 mm Hg (SD 7·51) at the 2-month visit. IOP lowering in the SLT group at the 2-month visit was 6·28 mm Hg (SD 6·13). To our knowledge, no previous direct comparison has been made between SLT and timolol eye drops, the most affordable and commonly available IOP lowering drug.^26^, ^30^

Gazzard and colleagues^13^ compared SLT with any conservative treatment to reduce IOP in a predominantly White study population in the UK. The authors followed an algorithm to define individual target IOPs and progression rules. Of the 536 eyes treated with SLT first, 419 (78%) required no additional medication to reach target IOP, and 321 (60%) required only a single SLT treatment.^13^ Realini and colleagues^31^ reported a study of 72 participants from an African Caribbean population with a 12-month success rate of 78%, using a 20% reduction from baseline IOP as success criterion.

Our adjusted multivariable model showed that a more advanced stage of glaucoma, higher baseline IOP, and the presence of exfoliation glaucoma were all associated with a decreased probability of success. In our study protocol, the stage of glaucoma determined the target IOP, which needs to be lower in advanced glaucoma.^2^ A greater reduction in IOP is more difficult to achieve in general; therefore, the probability of success is likely to be lower in eyes with advanced glaucoma and a higher baseline IOP (appendix 4 p 7) than in those with moderate glaucoma and a lower baseline IOP. Exfoliation glaucoma reduced the probability of success in both intervention groups. To date, few clinical trials with small sample sizes have shown inconclusive results concerning the role of exfoliation glaucoma.^32^ Our results suggest that, although the subtype of exfoliation glaucoma is challenging to treat overall, SLT might still be a better option than timolol (appendix 4 p 7). Some regions in sub-Saharan Africa are affected by a particularly high prevalence of exfoliation glaucoma.^33^

Only mild adverse effects and no serious treatment-related adverse events were reported in either group, similarly to other studies.^13^ SLT caused reversible changes in the anterior chamber and corneal endothelium, as well as no or mild pain in most patients.^34^ After excluding patients with asthma and bradycardia, timolol eye drops caused no clinically significant complaints. The extensive counselling by two Tanzanian research assistants probably played an important role in the high acceptance of both treatment methods, which could have possibly been lower otherwise. This trusting relationship and the provision of treatment at no cost probably contributed to the higher adherence to timolol eye drops in this trial compared with that observed in other studies.^22^, ^35^

There was no significant difference in preserving visual function or vision-related quality of life between the two groups. Gazzard and colleagues^13^ compared conservative treatment with SLT for patients with newly diagnosed glaucoma, in which general quality of life was the primary outcome. The trial did not find a difference in quality of life between the two intervention groups.

Besides the superior efficacy, comparable safety, and acceptance of SLT, cost is also an important factor. Out-of-pocket payment is still common in many countries and, even if national health insurance options are available, uptake might still be low.^15^ If an eye care unit uses SLT to treat at least 500 eyes with glaucoma per year, SLT laser therapy can be offered for around US$12·50, including estimates for salaries, cost of repair, and maintenance. The cost of repairing imported equipment can be high in regions where specialised service personnel sometimes need to be flown in or the equipment needs to be shipped abroad for maintenance or repair.^36^ The salaries of ophthalmologists and other eye care professionals are a crucial component. Both treatments can be offered as an affordable intervention for glaucoma using the annual gross domestic product per capita as a surrogate for income and an affordability threshold of 2·5%.^26^

Our trial has several limitations. To establish the IOP-lowering effect or the efficacy as accurately as possible, adherence to regular follow-up visits and eye drops was promoted through intensive counselling, phone call reminders, and subsidies for travel and treatment expenses. Although these efforts resulted in high follow-up rates, they are also a limitation of the study given that the results probably underestimate the difference between laser and eye drops, favouring timolol through the provision of free treatment, more intensive counselling, and transport support. Eye drops need to be applied daily and new bottles need to be purchased every few weeks for consistent IOP control. By contrast, SLT treatment requires only occasional IOP measurements and retreatments, if the IOP increases. SLT was consistently performed by one experienced eye surgeon, which assisted in determining the best possible efficacy of the procedure; however, such efficacy might not always be achieved, especially while eye care professionals are in their learning curve. A further limitation is the follow-up of 1 year. Although 1 year is a sufficient period to estimate the IOP-lowering potential of the interventions in our cohort, changes in visual outcomes, vision-related quality of life, long-term effects on IOP lowering, and the progression of glaucoma might only become apparent over a longer period of time. Longer follow-up would also allow target IOPs to be evaluated on and adjusted for particular eyes if necessary. Treatment affordability and cost were used to compare the two treatment alternatives, which is of particular relevance in regions with a high proportion of out-of-pocket payments. However, more comprehensive economic evaluations, such as an extended cost-effectiveness analysis that adds non-health benefits, including the financial risk protection and distributional consequences (eg, equity), are also particularly relevant in these regions and should be considered in future studies. Furthermore, it could be argued that alternative topical treatments, such as prostaglandin analogues, might have been more effective than timolol. However, our choice was deliberate because timolol is the current standard of care in the region, and such alternatives are either unavailable or prohibitively expensive.^8^

The target threshold of 18 mm Hg for advanced glaucoma was informed by the associative analysis of the AGIS trial, which found this threshold to be protective against further progression during a follow-up period of 6 years. It is noteworthy that AGIS also included patients with low baseline IOP, whereas our study enrolled patients with high-pressure glaucoma only (IOP >21 mm Hg).

The results from this trial suggest that SLT can be used instead of timolol eye drops, the current first-line treatment in sub-Saharan Africa. If glaucoma progresses further, SLT can be repeated or combined with eye drops before resorting to trabeculectomy, which remains an important treatment option for patients with glaucoma. Additionally, if surgeons are not confident in performing trabeculectomy (eg, in patients with end-stage glaucoma or when patients refuse surgery), SLT could have an important role. The initial investment cost can be offset, in this context, by completing around 500 procedures per year over 10 years. The laser treatment option could be embedded in a comprehensive glaucoma management network strategy based around large eye units equipped with an SLT laser. This strategy would need to be closely associated with improving community awareness, enabling early detection of glaucoma in primary care settings, and strengthening the referral pathways to these large eye units. Such an approach could increase the demand for affordable and convenient glaucoma treatment options, such as SLT.^15^

In summary, the prevalence of glaucoma is set to increase due to ageing and population growth, mainly in resource-limited settings.^5^, ^37^, ^38^ The *Lancet Global Health* Commission on global eye health suggested that research action is urgently needed to develop contextually relevant management strategies for glaucoma.^38^ The findings from this trial clearly indicate that SLT is superior to timolol eye drops in controlling IOP in patients with open-angle glaucoma in Tanzania. Both interventions showed similar safety profiles, acceptance by patients, vision-related quality of life, and preservation of visual acuity. Depending on the number of procedures and the funding model, SLT treatment can be offered at a similar cost to a 1-year supply of timolol eye drops. Ultimately, this trial, completed in Africa, provides strong evidence that SLT can contribute to an affordable management strategy for preventing blindness from glaucoma.

## Data sharing

The National Institute for Medical Research in Tanzania requires that all data sharing requests are reviewed and approved by them before data can be shared. Deidentified participant data is available to any researcher under reasonable request. To facilitate the data access process, please contact ethics@lshtm.ac.uk. The study protocol and statistical analysis plan are available from the corresponding author under reasonable request.

## Declaration of interests

GG reports personal fees from Alcon, Allergan, Belkin, Equinox, Genentech–Roche, Glaukos, Ivantis, Reichert, Sight Sciences, and from Thea; grants from Belkin, Santen, and from Thea; and non-financial involvement with the patient advocacy group GlaucomaUK, outside the submitted work; he is also a co-investigator on three other major SLT trials (LIGHT, COAST, and Belkin laser). All other authors declare no competing interests.

## Supplementary Materials

Kiswahili translation of the abstract

French translation of the abstract

Portuguese translation of the abstract
