# Supplementary material for: Selective laser trabeculoplasty versus 0·5% timolol eye drops for the treatment of glaucoma in Tanzania: a randomised controlled trial
Source: Lancet Glob Health. 2021 Oct 13;9(11):e1589–99. doi: 10.1016/S2214-109X(21)00348-X (PMC8526362; doi:10.1016/S2214-109X(21)00348-X)
Supplement: Kiswahili translation of the abstract [file mmc1.pdf]

THE LANCET
Global Health

Supplementary appendix 1

This translation in Kiswahili was submitted by the authors and we reproduce it as supplied. It has not been peer reviewed. *The Lancet*'s editorial processes have only been applied to the original in English, which should serve as reference for this manuscript.

Tafsiri hii katika Kiswahili iliwasilishwa na waandishi na tunatengeneza tena kama hutolewa. Haijapitiwa. Mchakato wa hariri wa *Lancet* umetumika tu kwa asili kwa Kiingereza, ambayo inapaswa kutumika kama kumbukumbu kwa muswada hii.

Supplement to: Philippin H, Matayan E, Knoll KM, et al. Selective laser trabeculoplasty versus 0·5% timolol eye drops for the treatment of glaucoma in Tanzania: a randomised controlled trial. *Lancet Glob Health* 2021; published online Oct 13. http://dx.doi.org/10.1016/S2214-109X(21)00348-X.

Selective Laser Trabeculoplasty na timolol 0·5% kwa ajili ya matibabu ya shinikizo la maji ya jicho nchini Tanzania: jaribio ya kulinganisha

Muhtasari

Usuli

Shinikizo la maji ya macho ni sababu kuu ya upotevu wa uoni duniani kote, hali inatokea zaidi barani Afrika. Tiba ya kawaida na rahisi inayotumika kudhibiti ugonjea huu ni dawa ya matone aina ya timolol ambayo hutumika kwa muda mrefu. Hata hivto uzingatiaji wa matibabu ni changamoto kubwa.

Utafiti ulikuwa na lengo la kuchunguza kama matibabu ya mionzi ya laser kudhibiti shinikizo la maji ya macho, ijulikanayo kiingereza kama Selective Laser Trabeculoplasty (SLT), ni bora zaidi kuliko dawa ya matone aina ya timolol.

Njia iliyotumika.

Jaribio hili la kitafiti lililofanyiaka Idara ya Macho katika Hospitali ya rufaa ya Kilimanjaro Christian Medical Centre, Moshi, Tanzania, liliweka makundi mawili sambamba bila ya washiriki kuchagua aina ya matibabu na pasipo daktari kujua matibabu yatakayotolewa. Washiriki walikuwa wana umri wa miaka ≥18, walikuwa na shinikizo la maji ya macho (Primary Open Angle Glaucoma), walikuwa na shinikizo la zaidi ya 21mmHg, hawajawahi kufanyiwa upasuaji wa jicho ili kushusa shinikizo, hawajawahi kupata matibabu ya SLT ya kudhibiti shinikizo la maji ya macho na wasio na pumu. Waliwekwa kwenye moja ya makundi mawili bila ya kuchagua kwa uwiano wa 1:1, kundi moja walipewa dawa ya matone ya timolol 0·5% ambayo walitumia mara mbili kwa siku na kundi la pili walifanyiwa matibabu ya SLT ya kudhibiti shinikizo la maji ya jicho. Matokeo ya makuu yalikuwa ni uwiano kati ya macho ya wale waliopatiwa tiba kuonyesha mafanikio miezi 12 baada ya kuingia kwenye utafiti, ambapo vigezo vya ufanisi zilikuwa ni shinikizo la maji ya macho chini au sawasawa na lengo lililowekwa kutokana na uharabifu uliosababishwa na ugonjwa. Kurudiwa tena kutoa maelekezo ya matumizi sahihi ya dawa za matone au kurudiwa kwa matibabu ya SLT iliruhusiwa mara moja tu. Uchambuzi wa awali ulikuwa na nia ya kufanya matibabu kwa kutumia vifaa na wale ambao hawakufuatilia matibabu waliondolewa. Uwiano wa jumla wa makadirio ulitumiwa kurekebisha uwiano kati ya macho. Jaribio hili liliandikishwa kwa Msajili wa Majaribio ya Kliniki ya Afrika (Pan African Clinical trial registry) kwa namba PACTR201508001235339.

Matokeo

Washiriki 201 (macho stahiki 382) waliandikishwa kutokana na wagonjwa 840 waliochunguzwa kati ya 31 Mei 2015 na 12 Mei 2017 ; watu 100 (50% ya washiriki, macho 191) bila mpangilio walipewa dawa ya macho ya timolol, na wengine 101 (50%, macho 191) walifanyiwa matibabu ya SLT.

Baada ya mwaka mmoja macho 339 yalifanyiwa utafiti (89%). Matibabu yalionyesha mafanikio kwenye macho 55/176 (31%) kwenye kundi la matone ya timolol, (macho 16/55 [29%] yalihitaji ushauri nasaha kwa mara ya pili kuhusu matumizi ya dawa) na macho 99/163 (61%) kwenye kundi la SLT (macho 33/99 [33%] yalihitajika kurudia SLT); odds ratio 3·37 (95% CI 1·96-5·80, p<0·0001). Madhara (yasiyohusu macho) yalitokea kwa washiriki 10 (10·%) waliokuwa kwenye kundi la dawa za matone ya timolol na nane (8%) waliokuwa kwenye kundi la SLT (p=0·61).

Tafsiri

SLT ilionesha ubora kuliko dawa ya matone ya timolol katika kudhibiti wagonjwa wenye shinikizo la maji ya macho kwa muda wa mwaka mmoja nchini Tanzania. Imeonyesha pia uwezo wa kubadilisha udhibiti wa shinikizo la maji ya macho kwenye mazingira ya Afrika Kusini mwa Sahara, hasa ambapo ugonjwa huu umeenea zaidi.

Ufadhili

CBM, Seeing is Believing Innovation Fund, na Wellcome Trust (207472/Z/17/Z).
