# Supplementary material for: Selective laser trabeculoplasty versus 0·5% timolol eye drops for the treatment of glaucoma in Tanzania: a randomised controlled trial
Source: Lancet Glob Health. 2021 Oct 13;9(11):e1589–99. doi: 10.1016/S2214-109X(21)00348-X (PMC8526362; doi:10.1016/S2214-109X(21)00348-X)
Supplement: French translation of the abstract [file mmc2.pdf]

THE LANCET
Global Health

Supplementary appendix 2

This translation in French was submitted by the authors and we reproduce it as supplied. It has not been peer reviewed. *The Lancet*'s editorial processes have only been applied to the original in English, which should serve as reference for this manuscript.

Cette traduction en français a été proposée par les auteurs et nous l'avons reproduite telle quelle. Elle n'a pas été examinée par des pairs. Les processus éditoriaux du *Lancet* n'ont été appliqués qu'à l'original en anglais et c'est cette version qui doit servir de référence pour ce manuscrit.

Supplement to: Philippin H, Matayan E, Knoll KM, et al. Selective laser trabeculoplasty versus 0·5% timolol eye drops for the treatment of glaucoma in Tanzania: a randomised controlled trial. *Lancet Glob Health* 2021; published online Oct 13. http://dx.doi.org/10.1016/S2214-109X(21)00348-X.

Trabéculoplastie sélective au laser versus 0·5% timolol en gouttes ophtalmiques pour le traitement du glaucome en Tanzanie : un essai contrôlé randomisé

Sommaire

Contexte

Le glaucome est une cause majeure de perte de la vue dans le monde entier ; la plus haute prévalence et incidence régionales ont été rapportées en Afrique. Le traitement à faible coût le plus couramment utilisé pour contrôler le glaucome est l'application à long terme du collyre timolol. Cependant, le manque d'observation du traitement par le patient constitue un défi majeur. Notre objectif consistait à déterminer si la trabéculoplastie sélective au laser (SLT) était supérieure aux gouttes ophtalmiques de timolol pour contrôler la pression intraoculaire (PIO) chez les patients atteints de glaucome à angle ouvert.

Méthodes

Nous avons réalisé un essai contrôlé et randomisé dans deux groupes, en mode parallèle et en simple insu, au service ophtalmique du Kilimanjaro Christian Medical Centre, à Moshi, en Tanzanie. Les participants éligibles (âgés de ≥18 ans) avaient un glaucome à angle ouvert et une PIO supérieure à 21 mm Hg. Ils ne souffraient pas d'asthme et n'avaient pas d'antécédents de chirurgie du glaucome ou de laser. Les participants ont été choisis au hasard (1 : 1) à recevoir soit des gouttes ophtalmiques de timolol 0·5% à administrer deux fois par jour, soit à recevoir une SLT. Le résultat principal était la proportion d'yeux traités avec succès dans chaque groupe ; le succès étant défini comme une PIO inférieure ou égale à la pression cible selon la gravité du glaucome, 12 mois après la randomisation. Une ré-explication de l'application des gouttes ophtalmiques ou une répétition de la SLT était autorisée une fois.
L'analyse primaire a été réalisée en intention de traiter modifiée, en excluant les participants perdus de vue, à l'aide d'une régression logistique ; des équations d'estimation généralisées ont été utilisées pour ajuster la corrélation entre les yeux. Cet essai a été enregistré dans le registre panafricain des essais cliniques, sous le numéro PACTR201508001235339.

Résultats

201 participants (382 yeux éligibles) ont été inclus parmi 840 patients dépistés entre le 31/08/2015 et le 12/05/2017 ; 100 personnes (191 yeux) ont été choisies de manière aléatoire pour le traitement timolol et 101 (191 yeux) pour la SLT. La PIO moyenne de départ était de 26·7mmHg (SD 6·9mmHg), 162 yeux avaient un glaucome modéré et 220 yeux avaient un glaucome avancé.
Après un an, 339 yeux ont été analysés (89%). Le traitement a été couronné de succès dans 55/176 yeux (31·3%) dans le groupe timolol (16/55 yeux ont nécessité une nouvelle consultation) et dans 99/163 yeux (60·7%) dans le groupe SLT (33/99 yeux ont nécessité une nouvelle SLT) ; odds ratio 3·37 (95% CI 1·96-5·80, p<0·0001). Des événements indésirables sont survenus chez 10 (10·0%) participants dans le groupe timolol et 8 (7·9%) dans le groupe SLT (p=0·61).

Interprétation

Dans la prise en charge des patients en Tanzanie atteints de glaucome à angle ouvert à haute pression intraoculaire, la SLT était supérieure aux gouttes ophtalmiques de timolol sur une période d'un an.
La SLT a le potentiel de transformer la prise en charge du glaucome en Afrique subsaharienne, même là où la prévalence du glaucome avancé est élevée.

Financement

CBM, Seeing is Believing Innovation Fund et Wellcome Trust (207472/Z/17/Z).
