# Supplementary material for: Selective laser trabeculoplasty versus 0·5% timolol eye drops for the treatment of glaucoma in Tanzania: a randomised controlled trial
Source: Lancet Glob Health. 2021 Oct 13;9(11):e1589–99. doi: 10.1016/S2214-109X(21)00348-X (PMC8526362; doi:10.1016/S2214-109X(21)00348-X)
Supplement: Portuguese translation of the abstract [file mmc3.pdf]

THE LANCET
Global Health

## Supplementary appendix 3

This translation in Portuguese was submitted by the authors and we reproduce it as supplied. It has not been peer reviewed. The Lancet's editorial processes have only been applied to the original in English, which should serve as reference for this manuscript.

Esta tradução em português foi submetida pelos autores e nós não fizemos quaisquer alterações. Esta versão não foi revista por pares. O processo editorial do The Lancet só foi aplicado à versão original em inglês, que deve servir como referência para este artigo.

Supplement to: Philippin H, Matayan E, Knoll KM, et al. Selective laser trabeculoplasty versus 0·5% timolol eye drops for the treatment of glaucoma in Tanzania: a randomised controlled trial. *Lancet Glob Health* 2021; published online Oct 13. http://dx.doi.org/10.1016/S2214-109X(21)00348-X.

# Trabeculoplastia laser selectiva versus 0·5% de colírio de timolol para o tratamento do glaucoma na Tanzânia: um ensaio aleatório controlado


## *Resumo*

**Contexto**

O glaucoma é uma das principais causas de perda de visão em todo o mundo, com a maior prevalência e incidência regional relatada em África. O tratamento de baixo custo mais comumente utilizado para controlar o glaucoma é o colírio de timolol a longo prazo. No entanto, a baixa aderência à terapêutica é um grande desafio. O nosso objectivo consistiu em investigar se a trabeculoplastia laser selectiva (TLS) era superior ao colírio de timolol para controlo da pressão intra-ocular (PIO) em doentes com glaucoma de ângulo aberto.

**Métodos**

Efectuou-se um ensaio clinico randomizado controlado de dois braços, cego, em grupo paralelo, no Departamento de Oftalmologia do Kilimanjaro Christian Medical Centre, Moshi, Tanzânia. Os participantes elegíveis (com idade ≥18 anos) tinham glaucoma de ângulo aberto e uma PIO acima de 21 mm Hg, e não tinham asma ou historial de cirurgia de glaucoma ou laser. Os participantes foram distribuídos aleatoriamente (1:1) para receberem 0·5% de colírio de timolol com administração duas vezes por dia ou para receberem TLS.

O resultado principal correspondeu à proporção de olhos de ambos os grupos com sucesso de tratamento, definida como uma PIO abaixo ou igual à pressão alvo, de acordo com a gravidade do glaucoma, 12 meses após a aleatorização. Permitiu-se a repetição da explicação da aplicação de gotas oftálmicas ou uma repetição do TLS, uma vez. A análise principal foi feita por intenção de tratamento modificada, excluindo os participantes perdidos para acompanhamento, usando regressão logística; foram usadas equações de estimativa generalizada para ajustar a correlação entre os olhos.

Este ensaio foi registado no Pan African Clinical Trials Registry, número PACTR201508001235339.

**Resultados**

840 pacientes foram examinados para elegibilidade, dos quais 201 (24%) participantes (382 olhos elegíveis) foram inscritos entre 31 de Agosto de 2015, e 12 de Maio de 2017. 100 (50%) dos participantes (191 olhos) foram distribuídos aleatoriamente pelo grupo timolol e 101 (50%; 191 olhos) pelo grupo SLT. Após 1 ano, foram analisados 339 (89%) de 382 olhos. O tratamento foi bem sucedido em 55 (31%) de 176 olhos no grupo do timolol (16 [29%] de 55 olhos requereram aconselhamento de administração repetida) e em 99 (61%) de 163 olhos no grupo do TLS (33 [33%] de 99 olhos requereram TLS repetida; odds ratio 3-37 [95% CI 1·96-5·80]; p<0·0001). Eventos adversos (na sua maioria não relacionados com eventos oculares) ocorreram em dez (10%) participantes do grupo timolol e em oito (8%) participantes do grupo TLS (p=0·61).

**Interpretação**

O TLS apresentou resultados superiores às gotas oftálmicas de timolol na gestão de pacientes com glaucoma de alta pressão de ângulo aberto durante 1 ano na Tanzânia. O TLS pode potencialmente transformar a gestão do glaucoma na África subsaariana, mesmo em locais onde a prevalência de glaucoma avançado é elevada.



**Financiamento**

CBM, Seeing is Believing Innovation Fund, e Wellcome Trust (207472/Z/17/Z).
