# Supplementary material for: Selective laser trabeculoplasty versus 0·5% timolol eye drops for the treatment of glaucoma in Tanzania: a randomised controlled trial
Source: Lancet Glob Health. 2021 Oct 13;9(11):e1589–99. doi: 10.1016/S2214-109X(21)00348-X (PMC8526362; doi:10.1016/S2214-109X(21)00348-X)
Supplement: Supplementary appendix 4 [file mmc4.pdf]

THE LANCET
Global Health

Supplementary appendix 4

This appendix formed part of the original submission and has been peer reviewed. We post it as supplied by the authors.

Supplement to: Philippin H, Matayan E, Knoll KM, et al. Selective laser trabeculoplasty versus 0·5% timolol eye drops for the treatment of glaucoma in Tanzania: a randomised controlled trial. *Lancet Glob Health* 2021; published online Oct 13. http://dx.doi.org/10.1016/S2214-109X(21)00348-X.

Selective laser trabeculoplasty versus 0·5% timolol eye drops for the treatment of glaucoma in Tanzania: a randomised controlled trial

Kilimanjaro Glaucoma Intervention Programme (KiGIP) – SLT trial

Supplementary appendix

Exclusion Criteria 2
Screening Results 3
Treatment Algorithm 4
Intraocular Pressure Results 5
Predicted Probabilities of Success 7
Interaction Plots 8
SLT Procedures: Transient Events 9
Cost Analysis 10
  Offering SLT treatment – the eye care provider's perspective 10
  Annual treatment cost for patients or health insurance companies 11
Affordability of Timolol Eye Drops and SLT Laser Procedure 11
Counselling of Patients Who Receive Timolol Eye Drops 12
  Purpose 12
  Procedure 12
References 14

Exclusion Criteria

Table A1: List of exclusion criteria

List of exclusion criteria
**Related to the eyes of the participant**
• Opaque cornea or anterior chamber which inhibits to visualise the chamber angle or to do SLT
• Narrow or closed chamber angle (less than Shaffer II in two out of four quadrants)
• No perception of light
• History of previous glaucoma surgery including other laser treatments
• History of previous uveitis
• Neovascular glaucoma
• Traumatic glaucoma
• Corneal irregularities affecting tonometry (e.g. advanced pterygium, astigmatism > 3dpt)
• Pseudophakic patients will be excluded if the chamber angle is blocked or the anterior segment shows signs of inflammation.
**Related to the participant**
• Inability to provide informed consent
• Unwillingness to return for regular follow-up visits (baseline + 4 follow-up visits, patients should come preferably from Arusha or Kilimanjaro Regions)
• Physical inability to administer topical treatment
• Age below 18 years.
• Pregnant women
• History of asthma, bradycardia, previous heart failure, hypersensitivity to beta-blockers

Screening Results

Table A2: Examination findings of all 840 patients with glaucoma screened for inclusion in this trial

		Total<br>N=840
Age	Mean (SD)	65·28 (14·65)
Sex	Female	351 (41·8%)
	Male	489 (58·2%)
Detailed screening results	Enrolled in trial	201 (23·9%)
	Had cyclophotocoagulation	22 (2·6%)
	Had selective laser trabeculoplasty	33 (3·9%)
	Had trabeculectomy	103 (12·3%)
	Had other glaucoma surgery	2 (0·2%)
	Secondary glaucoma	102 (12·1%)
	IOP ≤ 21mmHg	110 (13·1%)
	Ocular hypertension	8 (1·0%)
	Needs immediate surgery (e.g. trabeculectomy)	64 (7·6%)
	Narrow angle	10 (1·2%)
	Opaque cornea	19 (2·3%)
	Dense cataract	13 (1·5%)
	No perception of light	101 (12·0%)
	Has asthma	9 (1·1%)
	Didn't return for enrolment	8 (1·0%)
	Unable to return for regular follow-up	21 (2·5%)
	Refused to participate	13 (1·5%)
	Deceased before enrolment	1 (0·1%)

Data are presented as mean (SD) for continuous measures, and n (%) for categorical measures.

Treatment Algorithm

Figure A1: Treatment decision algorithm at follow-up examinations

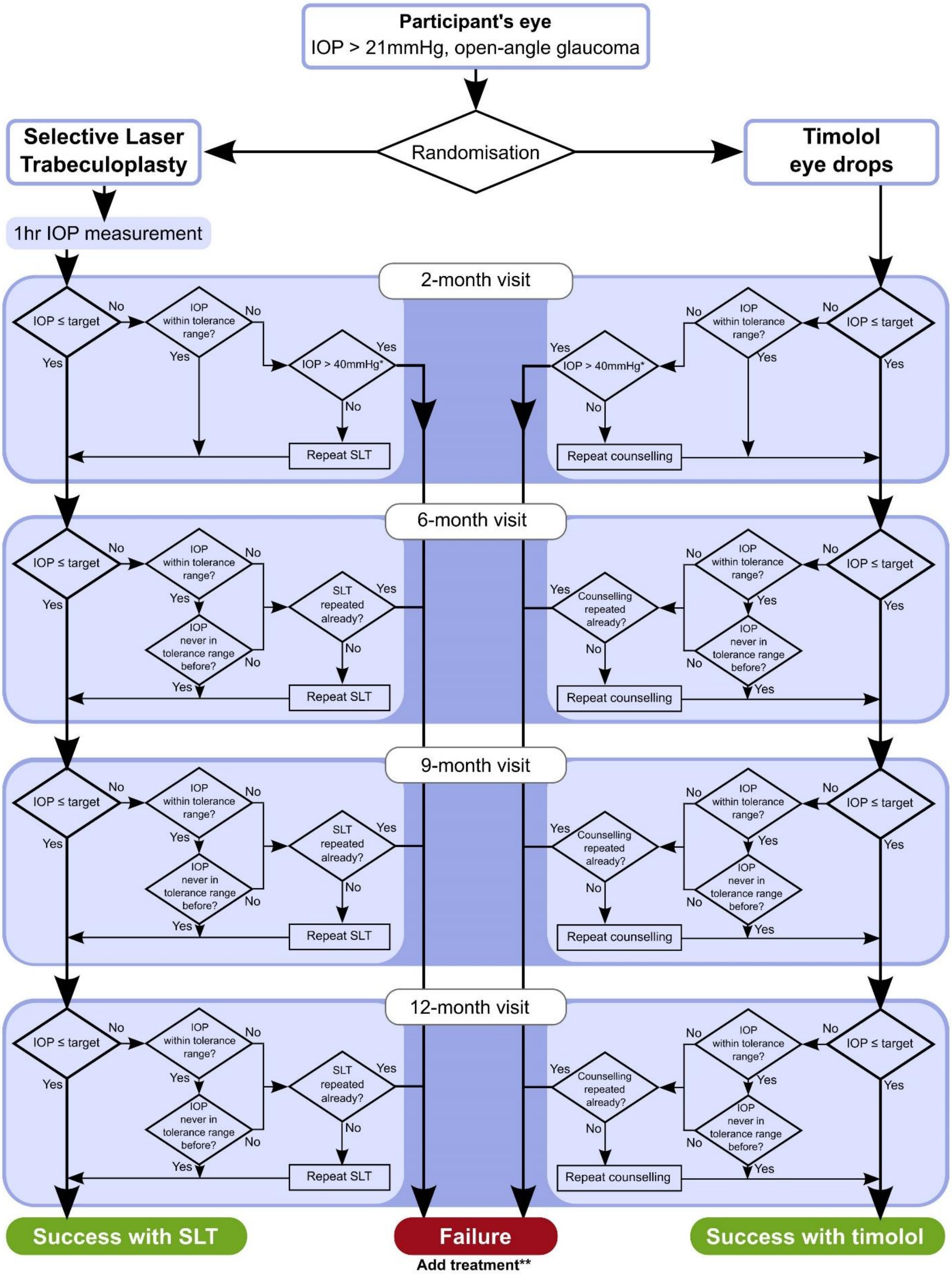

* IOP > 40mmHg at any follow-up visit is an immediate failure. For simplicity, the respective decision is only shown for the 2-month visit.
**Additional treatment will not be part of the trial and will be given according to clinical judgement of the treating ophthalmologist. However, standard additional treatment in the timolol group will be SLT and vice versa. Other addtional treatment options include latanoprost eye drops and trabeculectomy among others.

Intraocular Pressure Results

Table A3: Absolute IOP and relative IOP reductions at baseline, 2-month visit and study exit

	Timolol		SLT		All	
**Visit**	**n**	**mean (sd) / Δ (sd)**	**n**	**mean (sd) / Δ (sd)**	**n**	**mean (sd) / Δ (sd)**
Baseline	191	27·0 (7·5)	191	26·4 (6·3)	382	26·7 (6·9)
2-month	182	23·6 (7·4) /-3·2 (7·5)	181	20·0 (5·5) /-6·3 (6·1)	363	21·8 (6·8) /-4·7 (7·0)
Study exit	191	25·4 (7·8) /-1·5 (7·5)	191	20·1 (5·1) /-6·3 (6·4)	382	22·8 (7·1) /-3·9 (7·4)
Failure	121	28·5 (7·5) /-0·7 (8·9)	64	23·9 (4·6) /-5·5 (7·2)	185	26·9 (7·0) /-2·4 (8·6)
Loss to FU	15	25·5 (6·9) /-1·7 (2·7)	28	21·5 (6·5) /-3·1 (4·5)	43	22·9 (6·9) /-2·6 (4·0)
Success	55	18·6 (3·0) /-3·3 (4·1)	99	17·3 (2·7) /-7·7 (6·0)	154	17·8 (2·9) /-6·1 (5·8)

IOP - Intraocular pressure measurements (mmHg) at baseline and differences relative to baseline measurements at 2-month visits, failure visits, visits prior to loss to follow up or success visits at 1 year. Δ - delta or difference; SLT=selective laser trabeculoplasty; sd=standard deviation; FU=follow-up.

Table A4: Changes of intraocular pressure (%)

	Timolol		SLT		All	
**Visit**	**n**	**mean (sd) %**	**n**	**mean (sd)**	**n**	**mean % (sd)**
2-month	182	-9·2 (25·4)	181	-22·0 (18·8)	363	-15·6 (23·2)
Study exit	191	-2·8 (30·6)	191	-21·4 (20·0)	382	-12·1 (27·4)
Failure	121	2·6 (35·5)	64	-15·4 (20·8)	185	-3·6 (32·3)
Loss to FU	15	-6·2 (9·9)	28	-12·2 (17·1)	43	-10·1 (15·1)
Success	55	-13·6 (16·8)	99	-27·8 (18·0)	154	-22·7 (18·8)

Changes of intraocular pressure (%) relative to baseline measurements at 2-month visits; failure visits, visits prior to loss to follow up or success visits at 1 year. SLT=selective laser trabeculoplasty, sd=standard deviation, FU=follow-up.

Table A5: Intraocular pressure results at follow-up visits

	Timolol		SLT		All	
**Visit**	**n**	**mean (sd)**	**n**	**mean (sd)**	**n**	**mean (sd)**
Baseline	191	27·0 (7·5)	191	26·4 (6·3)	382	26·7 (6·9)
2-month	182	23·6 (7·4)	181	20·0 (5·5)	363	21·8 (6·8)
6-month	170	23·6 (7·1)	174	20·5 (4·4)	344	22·0 (6·1)
9-month	96	21·1 (3·8)	134	18·8 (3·5)	230	19·8 (3·8)
12-month	71	19·8 (4·0)	113	17·7 (2·9)	184	18·5 (3·5)

Intraocular pressure measurements of eyes (mmHg) at baseline and 2-month, 6-month, 9-month and 12-month visits including the respective study exit visit (failure, last visit before loss to follow-up or success at 12 months). SLT=selective laser trabeculoplasty, sd=standard deviation.

Figure A2: Boxplots of intraocular pressure results

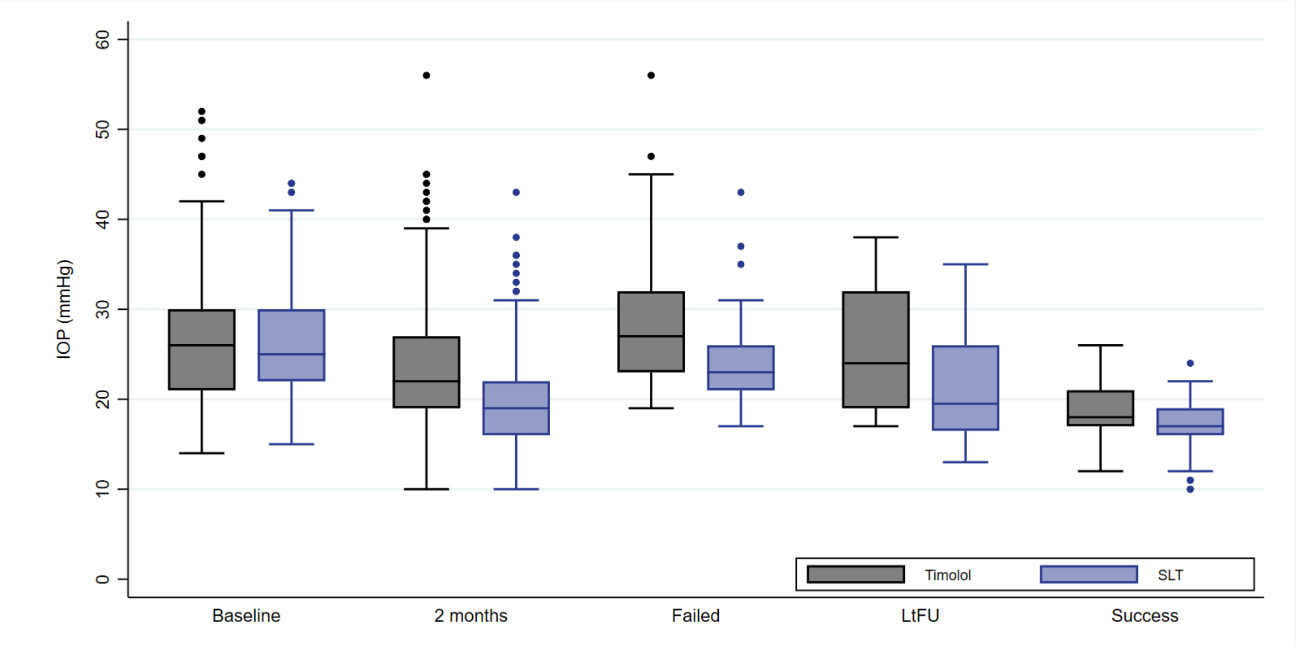

Boxplots of intraocular pressure measurements of the two treatment arms at baseline, 2-month, failure, before loss to follow-up, and success (at 12 months) visits. A box shows median, upper, and lower quartiles. Whiskers represent scores outside the middle 50%. Outliers are presented as individual dots. LtFU - loss to follow-up.

Predicted Probabilities of Success

Table A6: Predicted probabilities of success from the most parsimonious multivariable model

**Explanatory variables and levels**			**Timolol**			**SLT**		
			**Eyes n**	**Probability of success (95% CI)**	**p-value**	**Eyes n**	**Probability of success (95% CI)**	**p-value**
Stage of glaucoma	IOP at baseline	XFG						
Moderate	<25	No	41	0·72 (0·61, 0·84)	<0·001	44	0·93 (0·89, 0·98)	<0·001
		Yes	3	0·29 (0·05, 0·53)	0·016	3	0·69 (0·44, 0·93)	<0·001
	≥25	No	23	0·47 (0·32, 0·62)	<0·001	23	0·82 (0·73, 0·92)	<0·001
		Yes	4	0·12 (0·00, 0·24)	0·053	4	0·42 (0·15, 0·69)	0·002
Advanced	<25	No	28	0·22 (0·11, 0·33)	<0·001	27	0·61 (0·46, 0·75)	<0·001
		Yes	5	0·04 (0·00, 0·09)	0·106	2	0·19 (0·01, 0·38)	0·039
	≥25	No	62	0·09 (0·04, 0·14)	0·001	49	0·34 (0·22, 0·46)	<0·001
		Yes	10	0·01 (0·00, 0·03)	0·114	11	0·07 (0·00, 0·15)	0·059

Predicted probabilities of success derived from the most parsimonious multivariable model. XFG=exfoliation glaucoma. IOP=intraocular pressure, SLT=selective laser trabeculoplasty.

The probabilities of success as derived from the odds ratios of the multivariable model (see table 2 in the main manuscript) are shown in table A5. For example, eyes treated with SLT which had moderate glaucoma, a baseline IOP <25mmHg, and no exfoliation glaucoma had a higher probability of success (0·93, 95% CI 0·89-0·98, p<0.001) than eyes in the SLT arm affected by advanced glaucoma, a baseline IOP <25mmHg, and no exfoliation glaucoma (probability for success 0·61, 95% CI 0·46-0·75, p<0.001).

Interaction Plots

Figure A3: Interaction plot for intervention and stage of glaucoma

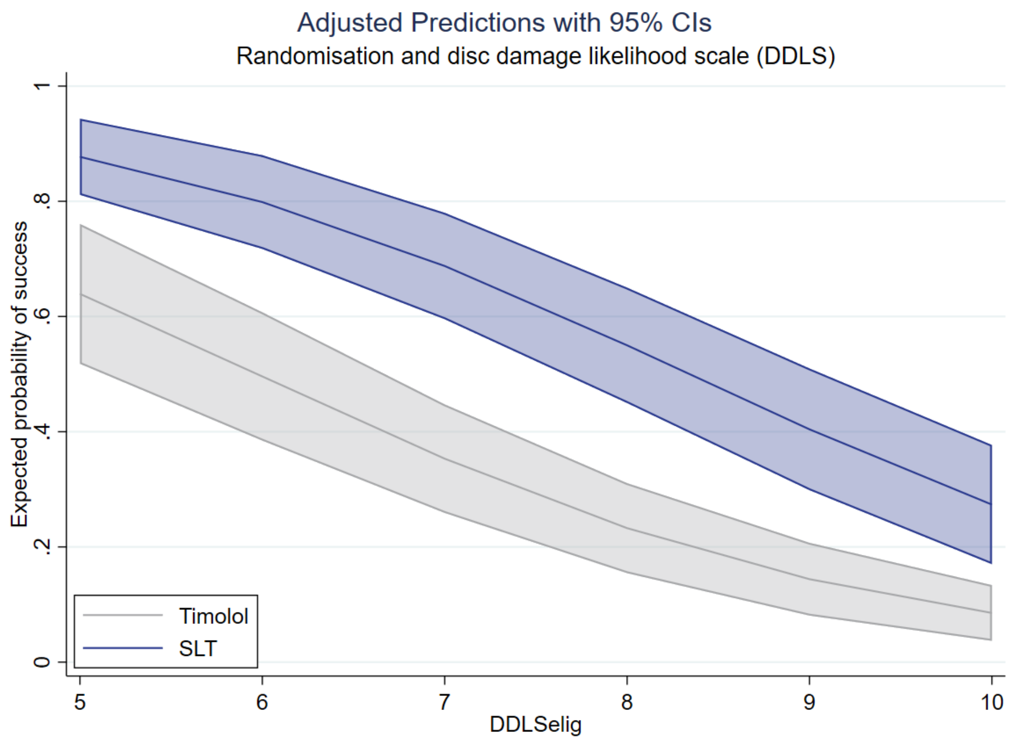

Figure A4: Interaction plot for intervention and baseline intraocular pressure

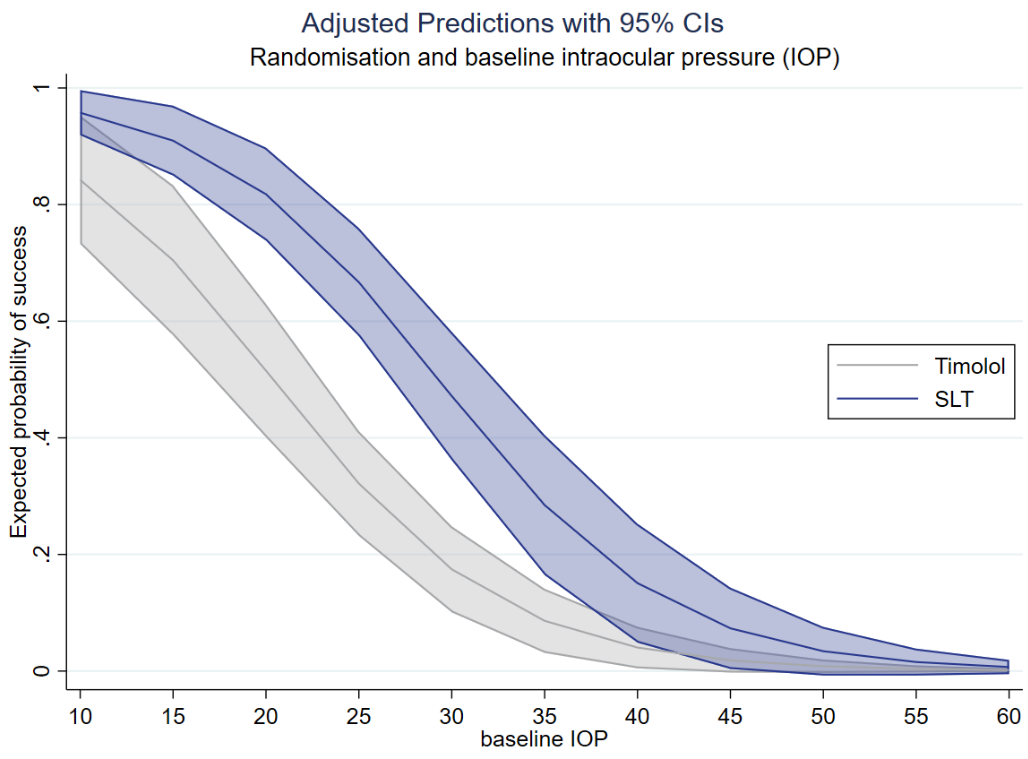

SLT Procedures: Transient Events

Table A7: Transient events during and shortly after SLT procedures

**Transient event**		**Baseline SLT N=191**	**Repeat SLT N=104**
Pain during SLT procedure*	no pain	69 (36·7%)	44 (44·0%)
	mild pain	103 (54·8%)	50 (50·0%)
	moderate pain	15 (8·0%)	6 (6·0%)
	severe pain	1 (0·5%)	0 (0%)
Cells in anterior chamber*	0·5+ (1-5)	49 (26·2%)	39 (38·6%)
	1+ (6-15)	129 (69·0%)	61 (60·4%)
	2+ (16-25)	9 (4·8%)	1(1·0%)
Endothelial changes**	No	114 (59·7%)	60 (58·3%)
	Translucent nummuli	77 (40·3%)	43 (41·7%)
IOP spike >5mmHg***	No	188 (100·0%)	99 (98·0%)
	Yes	0 (0·0%)	2 (2·0%)

Transient events during and after SLT procedures (within 1 hour), unit of reporting are eyes. Data are presented as n (%). Data missing for *seven eyes, **one eye, and ***six eyes. SLT=selective laser trabeculoplasty, IOP=intraocular pressure.

Cost Analysis

Offering SLT treatment – the eye care provider's perspective

Table A8: Assumptions, sources and measured parameters. All costs are reported in US Dollars

**Assumptions and measurements**			
Item	*Amount*	*Unit*	*Source*
Monthly ophthalmologist's salary	1600	$	Serje et al [1]
Working hours per month	160	hours	Gazzard et al [2]
SLT procedures done per hour (including preparations)	5	1/h	Data collected during trial
Price of brimonidine 0.2% eye drops (one bottle of 5ml)	5	$	KCMC Eye Department
Price of prednisolone 1% eye drops (one bottle of 5ml)	3	$	KCMC Eye Department
Price of amethocaine eye drops (one bottle of 5ml)	3	$	KCMC Eye Department
Number of eye drops in a 5ml bottle (approx.)	100	drops	Data collected during trial
No of preop. eye drops per SLT treatment (approx.)	4	drops	Data collected during trial
Annual rent (shared) and other fixed cost	700	$	Assumption
Maximum power consumption of SLT laser per hour	0·24	kWh	Operator manual [3]
Cost of 1 kWh	0·2	$	Assumption
Price of laser including contact glass and shipping	36000	$	CBM UK Finance Dept
Uninterruptible power supply (UPS), two replacement batteries	1200	$	Assumption
Lifespan of SLT laser	10	years	Gazzard et al [2]
Maintenance annual cost	300	$	Assumption
Repair cost including shipping of SLT laser	1500	$	Assumption
Maintenance interval[2]	1	year	Operator manual

We assumed that a sub-contracted local technician does the annual maintenance and for repairs the laser would need to be sent abroad. We assumed two repairs to be necessary during the expected life span of the SLT laser device of 10 years. Tanzanian Shilling (TZS) were converted to US$ using an average rate of 2,200 for the years 2015-2018. Duration of SLT treatment, average number of drops in an eye drop bottle and other parameters were determined as part of the study.

Table A9: Calculated cost items

	**A) Variable cost per procedure**	
Item	*Calculation*	*Cost (US$)*
Ophthalmologist's time	=($1600/ 160 hours) / 5 procedures per hour	2·0
Brimonidine eye drops	=($5 per bottle / 100 drops) * 4 drops	0·2
Prednisolone eye drops	=($3 per bottle / 100 drops) * 4 drops	0·12
Amethocaine eye drops	=($3 per bottle / 100 drops) * 8 drops	0·24
Power	=(240/1000 kWh * $0·2) / 5 procedures per hour	0·01
	**Total**	**2·57**

	**B) Fixed annual cost**	
Item	*Calculation*	*Cost (US$)*
SLT laser depreciation	=$36000 / 10 years	3,600
SLT laser maintenance	=$300 * 8 / 10 years	240
SLT laser repair	=$ 1500 *2 / 10 years	300
UPS	=$ 1200 / 10 years	120
Other (rent etc.)	=$ 700	700
	**Total**	**4,960**

Table A10: Total cost of a single SLT laser treatment for eight different hypothetical scenarios.

	Hypothetical annual number of treatments (n)							
	300	400	500	600	700	800	900	1000
Variable cost (US$)	2·57	2·57	2·57	2·57	2·57	2·57	2·57	2·57
Fixed cost (US$)	16·53	12·40	9·92	8·27	7·09	6·20	5·51	4·96
**Total cost per treatment (US$)**	**19·10**	**14·97**	**12·49**	**10·84**	**9·66**	**8·77**	**8·08**	**7·53**

Fixed cost per treatment calculated as total fixed annual cost/number of hypothetical annual treatments = 4,960/n US$.

Annual treatment cost for patients or health insurance companies

A) Timolol eye drops

The price per bottle of timolol (5ml) in Tanzania is approximately US$1·36. With 12 bottles per eye per year, the annual treatment cost amount to US$16·32. The cost of a 5ml timolol eye drop bottle was determined using the median of 3 prices at pharmacies across Tanzania in 2019.

B) SLT laser procedure

Assuming a hypothetical quantity of 500 eyes treated per year, the cost of a single SLT treatment is US$ 12·49 (table A4). According to this trial, an average of 1·33 SLT treatments is required for an eye to achieve successful IOP reduction. The resulting average treatment cost per year and per eye would be US$ 16·61 which is comparable to a supply of timolol eye drops for one year.

Affordability of Timolol Eye Drops and SLT Laser Procedure

Affordability describes whether a person has sufficient income to pay for health care services (or treatment costs). [4] It can be estimated using the average annual income of a person in need for treatment and an affordability threshold in relation to the cost of the treatment.[5,6]

We used the annual gross domestic product per capita as a surrogate for income and an affordability threshold of 2·5%.[5,6] The annual GDP per capita in Tanzania in 2019 was reported as US$1,122.12, so any annual treatment cost below US$28.05 should be considered affordable.[7]

Thus, the annual treatment cost of timolol and SLT for one eye are below this threshold (assuming 500 procedures per year in an eye health unit). For SLT, the treatment costs for two eyes can also be considered affordable for the majority of patients as 66/99 eyes (67%) only required one treatment to achieve a successful outcome (annual treatment cost for two eyes of US$24.98).

Counselling of Patients Who Receive Timolol Eye Drops

Purpose

This standard Operating Procedure) specifies the counselling of patients who are assigned to the conservative treatment arm with Timolol eye drops.

Adherence to topical treatment plays a pivotal role for the conservative management of glaucoma. Explaining the necessity of treatment and other means of motivation of patients can considerably improve adherence.[8,9]

Procedure

Overall Instructions

The talk will take place in a quiet environment and performed by a native Swahili speaker

Content

- Enquire about the level of knowledge and attitude
    - What do you know about your disease?
    - Which experience with any medical treatment do you have already? If any, what are the challenges for you (Cost? Side effects? Application? Understanding of mechanism?)
    - What are your concerns related to glaucoma and its treatment?
- Emphasize that treatment helps to prevent (further) loss of sight
- Mention known side effects of timolol eye drops: reduced libido, stinging, bradycardia, trouble breathing
- Explain application technique of eye drops or confirm who will apply eye drops (see below)

How to use and instil your own eye drops

1) How to open your eye drop bottle. ***Jinsi ya kufungua chupa yako ya matone ya macho.***

- Tight your bottle cap to the maximum end. ***Kaza chupa yako yenye matone mpaka mwisho kabisa.***
- Open and gently try to squeeze out a drop to see of the cap has punched a hole into the bottle tip. ***Taratibu fungua ili uone kama kifuniko kimetengeneza tundu kwenye mdomo wa chupa.***
- Use the same eye drop bottle for about one month before opening another one (in case you were given several bottles). ***Tumia chupa hiyo hiyo ya matone kwa kadri ya mwezi mmoja kabla ya kufungua nyingine. (ikiwa unazo chupa za ziada)***

2) How to instil your own eye drops **Jinsi ya kuweka matone kwenye macho yako.**

- Combine drop application time with your daily routine activities **Ambatanisha uwekaji wa matone na shughuli zako za kila siku, mfano; Kabla ya chai au chakula cha jioni**
- Sit or lie down with your head supported. As your skill develops you may eventually manage to instil your eye drops while standing. **Keti au lala na egemeza kichwa, kadri unavyotumia utazoea kuweka matone ukiwa umesimama.**
- Use your dominant hand to hold the bottle. **Shika chupa kwa mkono unaoutumia (kushoto au kulia)**
- With the index finger of your other hand, hold a clean piece of tissue or cotton wool (if available), and gently pull down the lower eyelid to form a 'pocket'. **Tumia kidole cha shahada kwa mkono mwingine na kitambaa kisafi,tishu au pamba kufungua jicho kwa chini**
- Hold the bottle between your thumb and forefinger, and place the 'heel' of your hand (where the wrist meets the hand) on your cheek. This will help to steady shaky hands. **Shika chupa yako kwa dole gumba na shahada, egemeza mkono juu ya uso kuzulia usitikisike.**
- Make sure there is a short distance of about an inch (2.5cm, the width of two fingers) between your eye and the end of the bottle. Be careful – the tip must not touch any part of the eye or eyelids. **Hakikisha unaacha nafasi kama inchi 2.5 kati ya vidole na uso na chupa ili usigusishe chupa yenye dawa na jicho lako au kope zako.**
- Look up or to the side. Do not look directly at the bottle. **Angalia juu au pembeni. Usiangalie chupa yenye matone.**
- Squeeze the bottle – allow one drop to fall into the lid pocket. **Binya chupa – hakikisha tone moja linaingia kwenye jicho ulilolifungua kwa kitambaa safi, tishu au pamba**
- Slowly let go of the lower lid. Gently close your eyes; try not to shut them tightly as this will squeeze the drop out of your eye. **Taratibu acha tone lisambae ndani ya jicho, funga jicho taratibu, usikaze jicho ili dawa isitoke nje.**
- Dab your closed eye with the tissue or cotton wool to remove any excess. **Ukiwa umefumba macho yako futa matone au machozi yaliyomwagika nje au pembeni ya macho kwa kitambaa safi, tishu au pamba**
- Put gentle pressure on the inside corner of your eye and count to 60, very slowly. This prevents the medicine from draining out of your eye before it is absorbed. **Kandamiza upande wa macho yako karibu na pua kwa dakika 1 au 2 ili kuzuia dawa kushuka kwenye koo lako na kubaki kwenye macho yako.**

(adapted from *Instilling your own eye drops.* Community Eye Health Journal. 2012; **79 & 80**: 79)

References

1 Serje J, Bertram MY, Brindley C, Lauer JA. Global health worker salary estimates: An econometric analysis of global earnings data. *Cost Eff Resour Alloc* 2018; **16**:10.

2 Gazzard G, Konstantakopoulou E, Garway-Heath D et al. Selective laser trabeculoplasty versus eye drops for first-line treatment of ocular hypertension and glaucoma (LiGHT): a multicentre randomised controlled trial. *Lancet* 2019; **393**: 1505–16.

3 Bara Bonnet. Selecta II Operator Manual. 2006 Lumenis, Israel.

4 Levesque JF, Harris MF, Russell G. Patient-centred access to health care: conceptualising access at the interface of health systems and populations. Int J Equity Health. 2013, **12**:18.

5 Niëns LM, Brouwer WBF. Measuring the affordability of medicines: Importance and challenges. *Health Policy* 2013; **112**:45–52.

6 Zhao PY, Rahmathullah R, Stagg BC et al. A Worldwide Price Comparison of Glaucoma Medications, Laser Trabeculoplasty, and Trabeculectomy Surgery. JAMA Ophthalmol. 2018; **136**:1271–9.

7 GDP per capita. World Development Indicators. 2019: https://data.worldbank.org/indicator/NY.GDP.PCAP.CD?locations=TZ (accessed 10/12/2020)

8 Sleath B, Blalock S, Covert D, Stone JL, Skinner AC, Muir K, et al. The Relationship between Glaucoma Medication Adherence, Eye Drop Technique, and Visual Field Defect Severity. Ophthalmology. 2011;**118**:2398–2402.

9 Sleath B, Blalock SJ, Carpenter DM, Sayner R, Muir KW, Slota C, et al. Ophthalmologist-patient communication, self-efficacy, and glaucoma medication adherence. Ophthalmology. 2015;**122**:748–54.

10 Instilling your own eye drops. Community Eye Health Journal. 2012; **79 & 80**: 79.
